# Interaction of multi-walled carbon nanotubes in mineral oil based Maxwell nanofluid

**DOI:** 10.1038/s41598-022-07958-y

**Published:** 2022-03-18

**Authors:** Hanifa Hanif, Sharidan Shafie

**Affiliations:** 1 Department of Mathematics, Sardar Bahadur Khan Women’s University, Quetta, Pakistan; 2 Department of Mathematical Sciences, Faculty of Science, Universiti Teknologi Malaysia, 81310 Johor Bahru, Johor Malaysia

**Keywords:** Fluid dynamics, Carbon nanotubes and fullerenes

## Abstract

The most pressing issue now is to improve the cooling process in an electrical power system. On the other hand, nanofluids are regarded as reliable coolants owing to their exceptional characteristics, which include excellent thermal conductivity, a faster heat transfer rate, and higher critical heat flux. Considering these fascinating properties of nanofluid, this research looks at the flow of mineral oil based Maxwell nanofluid with convective heat. Moreover, introducing heat radiation, viscous dissipation and Newtonian heating add to the novelty of the problem. The coupled partial differential equations supported by the accompanying boundary conditions are numerically solved using an implicit finite difference method. The simulations are carried out using MATLAB software, and the obtained results are illustrated graphically. It is observed that the velocity of fluid increases concernign the relaxation time parameter but decreases against fractional derivative.

## Introduction

The curiosity to explore the flow and heat transfer process in fluids obeying non-Newtonian rheological paradigms is increasing due to the extensive scope for industrial and technical applications; Fossil fuel burning, oil-well drilling, solidification processes, and material plasticizing for manufacturing parts are only a few examples. Over the last few decades, a number of approximate and self-consistent non-Newtonian rheological models have been presented, as no single model can include the characteristics of non-Newtonian fluids. Differential, rate, and integral models are the three types of models. In particular, rate-type fluid models such as Maxwell, Oldroyd-B, or Burgers fluids commonly forecast stress relaxation in polymer processing. Maxwell^1^ devised a fluid model to represent the elastic and viscous behavior of air at first. It is now commonly used to describe the behavior of a wide range of viscoelastic fluids, from polymers to the Earth’s mantle^2^.

Fractional calculus and its applications have grown in popularity and relevance over the last few decades, owing to its applicability in a wide range of scientific and technical fields. The fractional calculus is well-known for its suitability in describing memory effects and material hereditary features. In order to define frequency-dependent complex materials, fractional calculus was introduced to express the constitutive relation of Maxwell viscoelastic fluid. Moreover, fractional calculus theory has been utilized to describe thermoelasticity and heat conduction due to the global dependency of fractional operators^3^. Bagley and Torvik^4^ devised a link between molecular theories that predict the macroscopic behavior of certain viscoelastic media and an empirically developed fractional calculus approach to viscoelasticity. Makris et al.^5^ suggested a generalised Maxwell model with a fractional derivative, in which fractional-order derivatives supplanted the first-order derivatives of the Maxwell model. Moreover, the anomalous Maxwell model produces an algebraically decreasing stress relaxation modulus that agrees well with experimental data^6^. A time-fractional Maxwell fluid model has been numerically investigated by Hanif^7^. Jamshed addressed the entropy generation in Maxwell fluid flow in the presence of a magnetic field^8^.

In the last decade, the dispersion of nanoparticles in a base fluid has been considered to alter the characteristics of the fluid to optimize its thermal performance; Choi and Eastman initially proposed this idea^9^. The resulting fluid is coined as nanofluid in the literature^10–13^. Nanofluids have a wide range of applications, including pharmaceutical procedures, nano-drug delivery, heating/cooling appliances, microelectronics, nuclear power plants and fuel cells, to name a few. However, the effectiveness and application of a nanofluid depend on the nanoparticles suspended in the base fluid. Nanoparticles are a broad category of materials that include particulate substances with a minimum dimension of 100 nm, classified into several groups depending on their morphology, size, and chemical characteristics. Some of the most well-known classes of nanoparticles, based on physical and chemical features, include carbon-based, metal, ceramic, and semiconductor nanoparticles^14^. Carbon nanotubes (CNTs) are among the most common types of carbon-based nanoparticles and desirable materials in the manufacture of electrochemical devices because of their superior thermal and electrical conductivities, chemical and mechanical stability, featherweight, and consistency. Single-walled carbon nanotubes (SWCNTs) and multi-walled carbon nanotubes (MWCNTs) are two types of CNTs. In comparison to other nanoparticles, the heat conduction of nanofluids increased sixfold in the presence of CNTs^15^. In addition to that, CNTs have an extremely high thermal conductivity (2000–6000 W/m K), which is an order of magnitude higher than metallic or oxide nanomaterials like aluminum (237 W/m K) and aluminum oxide (40 W/m K), which are typically utilized as heat transfer enhancers in nanofluids^16^. Nanofluids with CNTs have substantially more excellent thermal properties such as heat transfer coefficient, thermal conductivity, and boiling heat flux when compared to their base fluids and nanofluids containing other types of nanomaterials^17–19^. The thermal management of electrical transformers is a critical in the electric power generating and distribution business to optimize system efficiency while maintaining safe operation. Oil, which might be synthetic or mineral, is used inside the electrical transformer to separate the internal components electrically. This oil also serves as a cooling medium for the transformer. In this perspective, increasing the oil dielectric strength and its capacity to transport heat from the transformer winding to the transformer shell via natural convection mechanisms might improve the performance of electrical transformers. Bearing this in mind, Fontes et al.^20^ experimented with analyzing the dynamic viscosity, thermal conductivity, and breakdown voltage of mineral oil based nanofluid for electrical transformers. According to their results, dynamic viscosity and thermal conductivity increased with increased particle concentration whereas breakdown voltage decreased with the addition of nanoparticles.

The purpose of this research is to scrutinize a two-dimensional flow of Maxwell nanofluid due to a constant pressure gradient. To the authors’ best knowledge, the interaction of MWCNTs inside mineral oil based Maxwell fluid with a sudden pressure force has not been investigated. Furthermore, when the qualities mentioned earlier are combined with thermal radiation, viscous dissipation, and Newtonian heating, this investigation becomes unique. The numerical solutions of the formulated model are generated in MATLAB using Crank–Nicolson finite difference assisted by time-fractional Caputo derivative.

## Governing equations

The Maxwell model depicts one of the most basic linear viscoelastic fluids with the constitutive equation:

1 $$\begin{aligned} {\mathbb {T}}=-\,{\mathbb {P}}{\mathbb {I}}+{\mathbb {S}}, \end{aligned}$$

where ${\mathbb {T}}$ is the well known Cauchy stress tensor, ${\mathbb {P}}$ is the hydro-static pressure, ${\mathbb {I}}$ is the identity tensor and ${\mathbb {S}}$ is the extra stress tensor, defined by the relationship:

2 $$\begin{aligned} {\mathbb {S}}+\lambda\bigg (\dfrac{\partial V}{\partial t}+V \cdot \nabla V-(\nabla V){\mathbb {S}}-{\mathbb {S}}(\nabla V)^{\dagger }\bigg )=\mu \bigg (\nabla V+(\nabla V)^{\dagger }\bigg ). \end{aligned}$$

Here *V* is the velocity field, $\mu$ is the dynamic viscosity, and $\lambda$ is the relaxation time parameter. We shall also define the extra stress tensor ${\mathbb {S}}$

3 $$\begin{aligned} {\mathbb {S}}= \begin{pmatrix} s_{xx}&{}\quad s_{xy}&{}\quad s_{xz}\\ s_{yx}&{}\quad s_{yy}&{}\quad s_{yz}\\ s_{zx}&{}\quad s_{zy}&{}\quad s_{zz} \end{pmatrix}. \end{aligned}$$

Fractional calculus has been successfully applied to the description of complicated dynamics such as relaxation, wave, and viscoelastic behavior in recent decades. Replacing the first derivative in the Maxwell fluid model with a fractional derivative of order $\alpha$ is a simple approach for adding fractional derivatives into models of linear viscoelasticity. Hence the fractional form of constitutive relation (2) can be written as:

4 $$\begin{aligned} {\mathbb {S}}+\lambda ^{\alpha }\bigg (\dfrac{\partial ^{\alpha } V}{\partial t^{\alpha }}+V \cdot \nabla V-(\nabla V){\mathbb {S}}-{\mathbb {S}}(\nabla V)^{\dagger }\bigg )=\mu \bigg (\nabla V+(\nabla V)^{\dagger }\bigg ). \end{aligned}$$

where $\dfrac{\partial ^{\alpha } V}{\partial t^{\alpha }}$ is Caputo time-fractional derivative with order $\alpha$ such that $0\le \alpha <1$. Following definition is required in the sequel.

**Definition 1 **Let ${\mathfrak {n}}\in {\mathbb {N}}$ and $\alpha \in {\mathbb {C}}$ with $\Re (\alpha )>0$ such that ${\mathfrak {n}}-1<\alpha <{\mathfrak {n}}$. Then for *f* in ${\mathbb {C}}^{{\mathfrak {n}}}({\mathbb {R}})$, the time-fractional Caputo derivative of order $\alpha$ is given by^21^:

5 $$\begin{aligned} \dfrac{\partial ^{\alpha } f(t)}{\partial t^{\alpha }}:= \dfrac{1}{\Gamma ({\mathfrak {n}}-\alpha )}\int _{0}^{t} (t-\tau )^{{\mathfrak {n}}-\alpha -1}\dfrac{\partial ^{\mathfrak {n}}}{\partial t^{\mathfrak {n}}}f(\tau )d\tau \end{aligned},$$

where $\Gamma (\cdot )$ is the gamma function, defined by

6 $$\begin{aligned} \Gamma (\eta )=\int _{\mathbb {R}}e^{-\psi } \psi ^{\eta -1}d\psi , \quad \forall \eta \in {\mathbb {C}} \quad {\text {such that}} \quad \Re (\eta )>0. \end{aligned}$$

The flow and heat transfer of an incompressible Maxwell nanofluid are governed by the following equations:

7 $$\begin{aligned}{}&\nabla \cdot V=0, \; \big ({\text {Continuity equation}}\big ) \end{aligned}$$

8 $$\begin{aligned}{}&\rho \bigg (\dfrac{\partial V}{\partial t}+V \cdot \nabla V\bigg )=\nabla .{\mathbb {T}}, \; \big ({\text {Momentum equation}}\big ) \end{aligned}$$

where $\rho$ is the density of the fluid.

9 $$\begin{aligned} \rho C_{p}\Bigg ( \dfrac{\partial T}{\partial t}+V\cdot \nabla T\Bigg )=k\Delta T+{\mathbb {T}}:\nabla V, \; \big ({\text {Energy equation}}\big ) \end{aligned}$$

here $C_{p}$ is the specific heat at constant pressure and *k* is the thermal conductivity.

### Problem description

Assume that the fluid is confined between two sidewalls of an infinite plate in the *xz*-plane. A pressure gradient is applied along the *x*-axis, which initiates the mainstream flow. As a consequence, the velocity field takes on the following form:

10 $$\begin{aligned} V=\big (u(y,z,t),0,0\big ), \end{aligned}$$

along with the extra stress tensor:

11 $$\begin{aligned} S=S(y,z,t). \end{aligned}$$

It is simple to verify that the velocity field of the aforementioned form meets the incompressibility criterion automatically. The momentum and energy Eqs. (8) and (9), respectively, reduced to

12 $$\begin{aligned}{}&\rho \dfrac{\partial u}{\partial t}=-\dfrac{\partial {\mathbb {P}}}{\partial x}+\dfrac{\partial s_{xy}}{\partial y}+\dfrac{\partial s_{xz}}{\partial z}. \end{aligned}$$

13 $$\begin{aligned}{}&\rho C_{p}\dfrac{\partial T}{\partial t}=k \bigg (\dfrac{\partial ^{2} T}{\partial y^{2}}+\dfrac{\partial ^{2} T}{\partial y^{2}}\bigg )+s_{xy}\dfrac{\partial u}{\partial y}+s_{xz}\dfrac{\partial u}{\partial z}. \end{aligned}$$

Furthermore, using Eqs. (4), (10) and (11), the extra stress tensor can be computed explicitly, yielding the relations:

14 $$\begin{aligned} s_{xy}+\lambda ^{\alpha }\dfrac{\partial ^{\alpha } s_{xy}}{\partial t^{\alpha }}=\mu \dfrac{\partial u}{\partial y}, \; {\text {and}} \; s_{xz}+\lambda ^{\alpha }\dfrac{\partial ^{\alpha } s_{xz}}{\partial t^{\alpha }}=\mu \dfrac{\partial u}{\partial z}, \end{aligned}$$

Multiplying Eq. (12) with $\bigg (1+\lambda ^{\alpha }\dfrac{\partial ^{\alpha }}{\partial t^{\alpha }}\bigg )$ and utilizing Eq. (14), we arrived at

15 $$\begin{aligned} \rho \bigg (1+\lambda ^{\alpha }\dfrac{\partial ^{\alpha }}{\partial t^{\alpha }}\bigg )\dfrac{\partial u}{\partial t}=-\bigg (1+\lambda ^{\alpha }\dfrac{\partial ^{\alpha }}{\partial t^{\alpha }}\bigg )\dfrac{\partial {\mathbb {P}}}{\partial x}+\mu \bigg (\dfrac{\partial ^{2} u}{\partial y^{2}}+\dfrac{\partial ^{2} u}{\partial z^{2}}\bigg ). \end{aligned}$$

In conjunction with thermal radiation, the energy Eq. (13) yields to

16 $$\begin{aligned} \rho C_{p}\dfrac{\partial T}{\partial t}=k \bigg (\dfrac{\partial ^{2} T}{\partial y^{2}}+\dfrac{\partial ^{2} T}{\partial y^{2}}\bigg )-\dfrac{\partial q_{r}}{\partial y}+s_{xy}\dfrac{\partial 
u}{\partial y}+s_{xz}\dfrac{\partial u}{\partial z}, \end{aligned}$$

where $q_{r}$ represents radiative heat flux and formulated by Rosseland approximation as^22,23^

17 $$\begin{aligned} q_{r}=-\dfrac{4 \sigma _{b}}{3 k_{b}}\dfrac{\partial T^{4}}{\partial y}. \end{aligned}$$

Here $\sigma _{b}$ is the Stefan–Boltzman coefficient and $k_{b}$ represents the absorption coefficient. Assume that the difference $T-T_{\infty }$ inside the flow domain is small enough such that $T^{4}$ can be expanded about $T_{\infty }$ using the Taylor series as

18 $$\begin{aligned} T^{4}\approxeq T^{4}_{\infty }+\dfrac{4T^{3}_{\infty}}{1!}\big (T-T_{\infty }\big )+\dfrac{12T^{2}_{\infty }}{2!}\big (T-T_{\infty }\big )^{2}+\ldots \end{aligned}$$

The higher orders are ignored since the temperature difference in the approximation is small enough, yielding in

19 $$\begin{aligned} T^{4}\approxeq T^{4}_{\infty }+4{T}^{3}_{\infty }\big (T-T_{\infty }\big )=4T^{3}_{\infty } T-3T^{4}_{\infty }. \end{aligned}$$

Invoking the approximation of $T^{4}$ in Eq. (17) results in^24^

20 $$\begin{aligned} q_{r}=-\dfrac{16\sigma _{b}T^{3}_{\infty }}{3k_b}\dfrac{\partial T}{\partial y}. \end{aligned}$$

Differentiating Eq. (20) w.r.t *y* and incorporating the resultant derivative in Eq. (16) yields to

21 $$\begin{aligned} {\begin{matrix} \rho C_{p}\dfrac{\partial T}{\partial t}&=\bigg (k+\dfrac{16\sigma _{b}}{3k_{b}}T^{3}_{\infty }\bigg )\dfrac{\partial ^{2} T}{\partial y^{2}}+k\dfrac{\partial ^{2}T}{\partial z^{2}}+s_{xy}\dfrac{\partial u}{\partial y}+s_{xz}\dfrac{\partial u}{\partial z}, \end{matrix}} \end{aligned}$$

### Flow and heat transfer modeling of nanofluid

There are several types of heat transfer modeling available today, including dispersion model, particle migration, single-phase and two-phase models^25^. In this research, we considered single-phase model by replacing traditional fluid's thermal and physical properties with the corresponding properties of nanofluid. Consequently, Eqs. (15) and (21) can be modified as

22 $$\begin{aligned}{}&\rho _{nf}\bigg (1+\lambda ^{\alpha }\dfrac{\partial ^{\alpha }}{\partial t^{\alpha }}\bigg )\dfrac{\partial u}{\partial t}=-\bigg (1+\lambda ^{\alpha }\dfrac{\partial ^{\alpha }}{\partial t^{\alpha }}\bigg )\dfrac{\partial {\mathbb {P}}}{\partial x}+\mu _{nf} \bigg (\dfrac{\partial ^{2} u}{\partial y^{2}}+\dfrac{\partial ^{2} u}{\partial z^{2}}\bigg ). \end{aligned}$$

23 $$\begin{aligned}{}&(\rho C_{p})_{nf}\dfrac{\partial T}{\partial t}=\bigg (k_{nf}+\dfrac{16\sigma _{b}}{3k_{b}}T^{3}_{\infty }\bigg )\dfrac{\partial ^{2} T}{\partial y^{2}}+k_{nf}\dfrac{\partial ^{2}T}{\partial z^{2}}+s_{xy}\dfrac{\partial u}{\partial y}+s_{xz}\dfrac{\partial u}{\partial z}. \end{aligned}$$

The fluid is initially at rest and at ambient temperature. Therefore, applying the following initial conditions is reasonable. For $t<0, \, (y,z) \in [0,\infty ) \times [0,z_{max}],$

24 $$\begin{aligned} u(y,z,t)=0=\dfrac{\partial u(y,z,t)}{\partial t}, \, T(y,z,t)=T_{\infty }. \end{aligned}$$

We impose a no-slip velocity and Newtonian heating conditions along the plate and the walls so that:

25 $$\begin{aligned} {\left\{ \begin{array}{ll} u(0,z,t)=0, \; \dfrac{\partial T(0,z,t)}{\partial y}=-h_{s}T, &{}\quad t>0,\, z \in [0,z_{max}],\\ u(y,0,t)=0=u(y,z_{max},t), &{}\quad t>0,\, y\in [0,\infty ),\\ T(y,0,t)=T_{\infty }=T(y,z_{max},t), &{}\quad t>0,\, y\in [0,\infty ). \end{array}\right. } \end{aligned}$$

The natural far field conditions are

26 $$\begin{aligned} u(y,z,t)\rightarrow 0, \; T(y,z,t)\rightarrow T_{\infty } \; {\text {as}}\; y\rightarrow \infty . \end{aligned}$$

Furthermore, the flow is propelled by a constant pressure gradient in the *x*-direction

27 $$\begin{aligned} \dfrac{\partial {\mathbb {P}}}{\partial x}=-p_{0}\rho _{f}{\mathbb {H}}(t), \end{aligned}$$

where ${\mathbb {H}}$ denotes the Heaviside function, which is defined as follows

28 $$\begin{aligned} {\mathbb {H}}(t)= {\left\{ \begin{array}{ll} 1, &{}\quad t\ge 0,\\ 0, &{}\quad t<0. \end{array}\right. } \end{aligned}$$

### Thermal and physical properties of nanofluid

Let $\varphi$ represents the volume fraction of nanomaterial, and the subscripts *f* and *nf* refer to base fluid and nanofluid, then thermal and physical properties of nanofluid are defined as follows

#### Density of nanofluid

The density of an object is defined as the mass divided by the volume of the object. The mathematical expression for density of nanofluid is given by^26,27^

29 $$\begin{aligned} \rho _{nf}\, {:}{=}\, \rho _{f}\big ((1-\varphi )+\varphi {\rho _{s}}/{\rho _{f}}\big ). \end{aligned}$$

#### Specific heat capacity of nanofluid

The amount of energy required to raise the temperature of 1 g of a substance by 1 $^{\circ }$C is known as the specific heat of a substance. The mathematical expression in terms of base fluid and nanoparticle is defined as

30 $$\begin{aligned} C_{P_{nf}}\, {:}{=}\, C_{p_{f}}\big ((1-\varphi )+\varphi C_{p_{s}}/C_{p_{f}}\big ). \end{aligned}$$

Later, Xuan and Roetzel amended this correlation by considering thermal equilibrium between nanomaterial and the liquid phase and modified the above equation by taking the density into account^28–30^

31 $$\begin{aligned} \big (\rho C_{P}\big )_{nf}\, {:}{=}\, \big (\rho C_{p}\big )_{f}\big ((1-\varphi )+\varphi \big (\rho C_{p}\big )_{s}/\big (\rho C_{p}\big )_{f}\big ). \end{aligned}$$

#### Thermal conductivity of nanofluid

The ability of a material to conduct heat is referred to as thermal conductivity. It is an essential factor for determining the heat transfer capacity in a thermal system. Well-known Maxwell theory for the effective thermal conductivity of a liquid with a dilute suspension of spherical particles is given as^31,32^

32 $$\begin{aligned} k_{nf}\, {:}{=}\, k_{f}\dfrac{k_{s}+2k_{f}+2\varphi (k_{s}-k_{f})}{k_{s}+2k_{f}-\varphi (k_{s}-k_{f})}. \end{aligned}$$

Later, Hamilton and Crosser extended Maxwell model by taking shape factor of nanoparticle into account. Their proposed model is^33–35^

33 $$\begin{aligned} k_{nf}\,{:}{=}\, k_{f}\dfrac{k_{s}+(n-1)k_{f}+(n-1)\varphi (k_{s}-k_{f})}{k_{s}+(n-1)k_{f}-\varphi (k_{s}-k_{f})}, \end{aligned}$$

where $n=3/\varpi$ is the shape factor with sphericity $\varpi$. Note that Hamilton Crosser model reduces to Maxwell model for $\varpi =1$. Xue^36^ claimed that the existing models are unable to reveal the influence of CNT space distribution on thermal conductivity. In general, CNTs are considered as rotating elliptical nanoparticles with a very large axial ratio, which ensures that current models might not work on CNT-based composites. According to Xue, the thermal conductivity for model CNT-based composites is

34 $$\begin{aligned} k_{nf}\, {:}{=}\, k_{f}\dfrac{1-\varphi +2\varphi \dfrac{k_{s}}{k_{s}-k_{f}}ln\dfrac{k_{s}+k_{f}}{2k_{f}}}{1-\varphi +2\varphi \dfrac{k_{f}}{k_{s}-k_{f}}ln\dfrac{k_{s}+k_{f}}{2k_{f}}}. \end{aligned}$$

#### Dynamic viscosity of nanofluid

Dynamic viscosity is a measure of internal resistance to flow. Einstein’s formula for calculating the effective viscosity $\mu _{nf}$ of a linearly viscous fluid with viscosity $\mu _{f}$ and a dilute suspension of small spherical particles is

35 $$\begin{aligned} \mu _{nf}\,{:}{=}\,\big (1+2.5\varphi \big )\mu _{f}. \end{aligned}$$

Einstein’s equation was extended by Brinkman^37,38^ as

36 $$\begin{aligned} \mu _{nf}\, {:}{=}\, \dfrac{\mu _{f}}{(1-\varphi )^{2.5}}. \end{aligned}$$

Xuan et al.^39^ performed an experiment to measure the apparent viscosity of the oil–water and water–copper nanofluid at temperatures ranging from 20 to 59 $^{\circ }$C. The findings of the experiment show that Brinkman’s theory is fairly accurate^28^.

**Table 1:** Thermal and physical properties of mineral oil and MWCNTs^20^.

Materials	Mineral oil	MWCNTs
$$\rho$$ (kg/m$$^{3})$$	861	2100
*k* (W/mK)	0.157	3000
$C_{p}$ (J/kg K)	1860	710
$\mu$ (Pa s)	0.01335	–

### Non-dimensional flow and heat transfer model

To comprehend the physics of the presented problem, non-dimensional representation is required. Therefore, we introduced the following non-dimensional parameters

37 $$\begin{aligned} \begin{aligned}{}&\bar{y}=\dfrac{y}{z_{max}}, \quad \bar{z}=\dfrac{z}{z_{max}}, \quad \bar{t}=\dfrac{\nu _{f} t}{z_{max}^{2}}, \quad \bar{u}=\dfrac{uz_{max}}{\nu _{f}},\\&\bar{T}=\dfrac{T-T_{\infty }}{T_{\infty }}, \quad \bar{\lambda }= \dfrac{\lambda \nu _{f}}{z_{max}^{2}}, \quad \bar{s}_{xy}=\dfrac{z_{max}^{2}s_{xy}}{\mu _{f}\nu _{f}}, \quad \bar{s}_{xz}=\dfrac{z_{max}^{2}s_{xz}}{\mu _{f}\nu _{f}}. \end{aligned} \end{aligned}$$

Incorporating the non-dimensional parameters (37) in Eqs. (22), (23), (24), (25) and (26) yield to following (after removing the bars for simplicity)

38 $$\begin{aligned}{}&\phi _{1}\bigg (1+\lambda ^{\alpha }\dfrac{\partial ^{\alpha }}{\partial t^{\alpha }}\bigg )\dfrac{\partial u}{\partial t}=p\bigg ({\mathbb {H}}(t)+\lambda ^{\alpha }\dfrac{t^{-\alpha }}{\Gamma (1-\alpha )}\bigg )+\phi _{2} \bigg (\dfrac{\partial ^{2} u}{\partial y^{2}}+\dfrac{\partial ^{2} u}{\partial z^{2}}\bigg ), \end{aligned}$$

39 $$\begin{aligned}{}&Pr\phi _{3}\dfrac{\partial T}{\partial t}=\bigg (\phi _{4}+Rd\bigg )\dfrac{\partial ^{2} T}{\partial y^{2}}+\phi _{4}\dfrac{\partial ^{2}T}{\partial z^{2}}+{\mathscr {E}}\biggl \{s_{xy}\dfrac{\partial u}{\partial y}+s_{xz}\dfrac{\partial u}{\partial z}\biggr \}, \end{aligned}$$

subject to the initial and boundary conditions

40 $$\begin{aligned} {\left\{ \begin{array}{ll} u(y,z,t)=0=\dfrac{\partial u(y,z,t)}{\partial t}, \; T(y,z,t)=0, \;t<0, \; (y,z) \in [0,\infty ) \times [0,z_{max}],\\ u(y,0,t)=0=u(y,z_{max},t), \; T(y,0,t)=0=T(y,z_{max},t), \; t>0,\; y\in [0,\infty ),\\ u(0,z,t)=0, \; \dfrac{\partial T(0,z,t)}{\partial y}=-\gamma \big (1+T(0,z,t)\big ), \; t>0,\; z \in [0,z_{max}],\\ u(y,z,t)\rightarrow 0, \; T(y,z,t)\rightarrow 0 \; {\text {as}}\; y\rightarrow \infty . \end{array}\right. } \end{aligned}$$

given that

41 $$\begin{aligned} & \phi _{1}=(1-\varphi )+\varphi {\rho _{s}}/{\rho _{f}}, \; \phi _{2}=\big (1-\varphi \big )^{-2.5}, \; \phi _{3}=(1-\varphi )+\varphi \big (\rho C_{p}\big )_{s}/\big (\rho C_{p}\big )_{f}, \; \gamma =h_{s}z_{max},\\ & \phi _{4}=\dfrac{1-\varphi +2\varphi \dfrac{k_{s}}{k_{s}-k_{f}}ln\dfrac{k_{s}+k_{f}}{2k_{f}}}{1-\varphi +2\varphi \dfrac{k_{f}}{k_{s}-k_{f}}ln\dfrac{k_{s}+k_{f}}{2k_{f}}}, \; p=\dfrac{p_{0}z_{max}^{3}}{\nu _{f}^{2}}, \; Rd=\dfrac{16\sigma _{b}}{3k_{b}k_{f}}, \;{\mathscr {E}}=\dfrac{\mu _{f}\nu _{f}^{2}}{k_{f}z_{maz}^{2}T_{\infty }}, \; Pr=\dfrac{\mu _{f} C_{p_{f}}}{k_{f}}.  \end{aligned}$$

## Numerical analysis

Define $t_{k}=k{\mathfrak {h}}, k=0, 1,\ldots ,{\mathfrak {n}}$, $y_{i}=i{\mathfrak {p}}, i=1,2,\ldots , {\mathfrak {r}}$, $z_{j}= j{\mathfrak {q}}, j=1,2,\ldots , {\mathfrak {s}}$, where ${\mathfrak {h}}=t_{\mathfrak {f}}/n$ is the time step, ${\mathfrak {p}}=y_{max}/{\mathfrak {r}}$ and ${\mathfrak {q}}=z_{max}/{\mathfrak {s}}$ are the mesh size in (*y*, *z*) direction. The discrete forms of Eqs. (38), (39) and (40) are obtained using the Crank–Nicolson method. It is a finite difference approach that is not only unconditionally stable, but also has fast convergence and accuracy^40^. Moreover, the fractional-time derivatives are evaluated using Caputo time-fractional derivative. The discrete momentum equation is

42 $$\begin{aligned} &\Bigg (\dfrac{\phi _{1}}{{\mathfrak {h}}}+\phi _{1}\lambda ^{\alpha }\dfrac{{\mathfrak {h}}^{-(\alpha +1)}}{\Gamma (2-\alpha )}\Bigg )\Bigg [u_{i,j}^{k+1}-u_{i,j}^{k}\Bigg ]\\&\quad =\dfrac{p_{0}}{2}\Bigg [{\mathbb {H}}(t_{k})+{\mathbb {H}}(t_{k+1})+\lambda ^{\alpha }\dfrac{t_{k}^{-\alpha }+t_{k+1}^{-\alpha }}{\Gamma (1-\alpha )}\Bigg ]+\dfrac{\phi _{2}}{2{\mathfrak {p}}^{2}}\Bigg [u^{k+1}_{i-1,j}-2u_{i,j}^{k+1}+u^{k+1}_{i+1,j}+u^{k}_{i-1,j} -2u^{k}_{i,j}+u^{k}_{i+1,j}\Bigg ]\\&\qquad +\dfrac{\phi _{2}}{2{\mathfrak {q}}^{2}}\Bigg [u^{k+1}_{i,j-1}-2u_{i,j}^{k+1}+u^{k+1}_{i,j+1}+u^{k}_{i,j-1}-2u^{k}_{i,j}+u^{k}_{i,j+1}\Bigg ]\\&\qquad+\phi _{1}\lambda ^{\alpha }\dfrac{{\mathfrak {h}}^{-(\alpha +1)}}{\Gamma (2-\alpha )}\sum _{{\mathfrak {m}}=1}^{k}{\mathfrak {b}}_{{\mathfrak {m}}}\Bigg [u_{i,j}^{k+1-{\mathfrak {m}}}-u_{i,j}^{k-{\mathfrak {m}}}\Bigg ], \end{aligned}$$

where $${\mathfrak {b}}_{{\mathfrak {m}}}=\big ({\mathfrak {a}}_{{\mathfrak 
{m}}}-{\mathfrak {a}}_{{\mathfrak {m}}-1}\big )$$ with $${\mathfrak {a}}_{{\mathfrak {m}}}=({\mathfrak {m}}+1)^{1-\alpha }-{\mathfrak {m}}^{1-\alpha }$$. The discrete form of Eq. (39) is given by

43 $$\begin{aligned}\dfrac{\phi _{3}Pr}{{\mathfrak {h}}}\Bigg [T_{i,j}^{k+1}-T_{i,j}^{k}\Bigg ]&=\dfrac{\big (\phi _{4}+Rd\big )}{2{\mathfrak {p}}^{2}}\Bigg [T^{k+1}_{i-1,j}-2T_{i,j}^{k+1}+T^{k+1}_{i+1,j}+T^{k}_{i,j+1}-2T^{k}_{i,j}+T^{k}_{i,j}\Bigg ]\\ &\quad +\dfrac{\phi _{4}}{2{\mathfrak {q}}^{2}}\Bigg [T^{k+1}_{i,j-1}-2T_{i,j}^{k+1}+T^{k+1}_{i,j+1}+T^{k}_{i,j-1}-2T^{k}_{i,j}+T^{k}_{i,j+1}\Bigg ]+ \dfrac{{\mathscr {E}} }{4{\mathfrak {p}}}\Bigg (s_{xy}(k+1)+s_{xy}(k)\Bigg )\Bigg [u_{i+1,j}^{k+1}-u_{i,j}^{k+1}+u_{i+1,j}^{k}-u_{i,j}^{k}\Bigg ]\\ {} &\quad +\dfrac{{\mathscr {E}}}{8 {\mathfrak {q}}}\Bigg (s_{xz}(k+1)+s_{xz}(k)\Bigg )\Bigg [u_{i,j+1}^{k+1}-u_{i,j-1}^{k+1}+u_{i,j+1}^{k}-u_{i,j-1}^{k}\Bigg ]. \end{aligned}$$

44 $$\begin{aligned}{}&{\begin{matrix} {\left\{ \begin{array}{ll} u_{i,j}^{0}=0=T_{i,j}^{0}, \; u_{0,j}^{k}=0,\\ T_{-1,j}^{k+1}+T_{-1,j}^{k}=2{\mathfrak {p}}\gamma \Big [2+T_{0,j}^{k+1}+T_{0,j}^{k}\Big ]+T_{1,j}^{k+1}+T_{1,j}^{k},\\ u_{i,0}^{k}=u_{i,{\mathfrak {s}}}^{k}=u_{{\mathfrak {r}},j}^{k}=0=T_{i,0}^{k}=T_{i, {\mathfrak {s}}}^{k}=T_{{\mathfrak {r}},j}^{k}. \end{array}\right. } \end{matrix}} \end{aligned}$$

**Figure 1:**
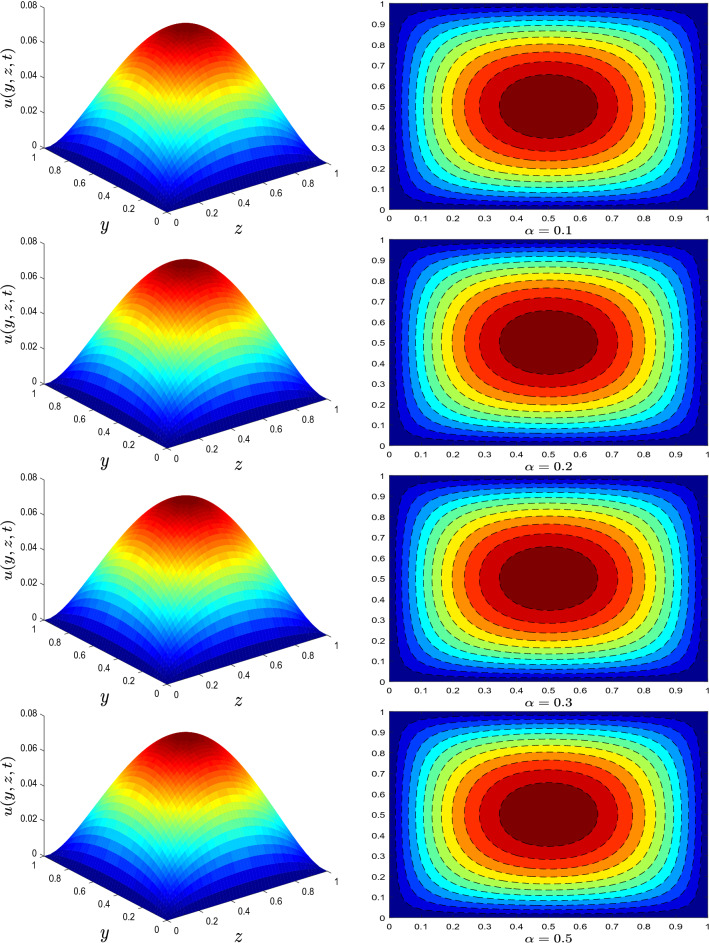
Velocity profile for different values of fractional derivative parameter $\alpha$.

**Figure 2:**
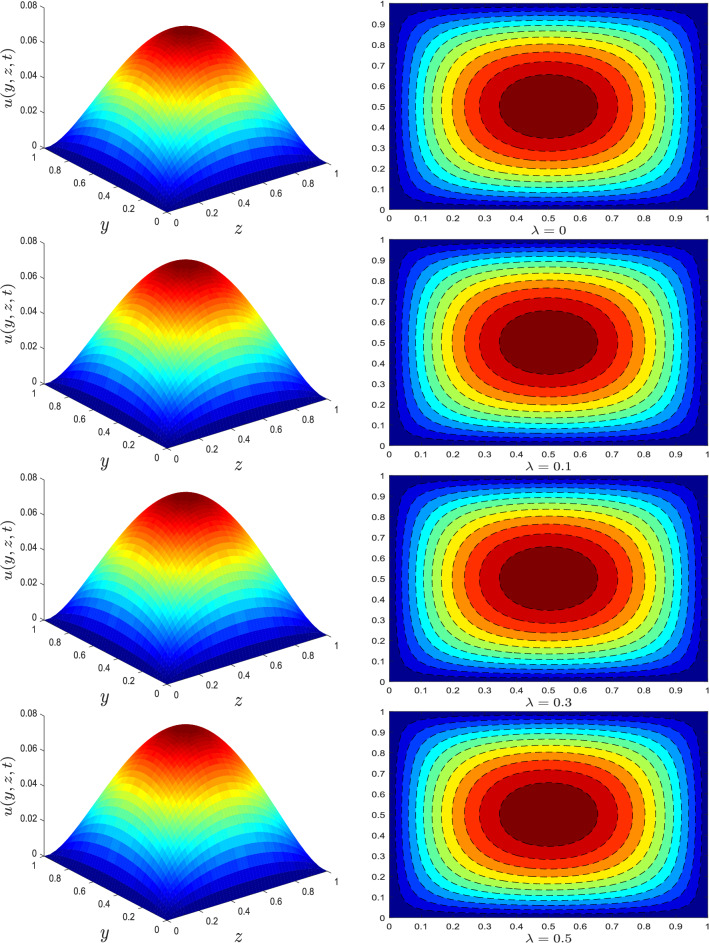
Velocity profile for different values of relaxation time parameter $\lambda$.

**Figure 3:**
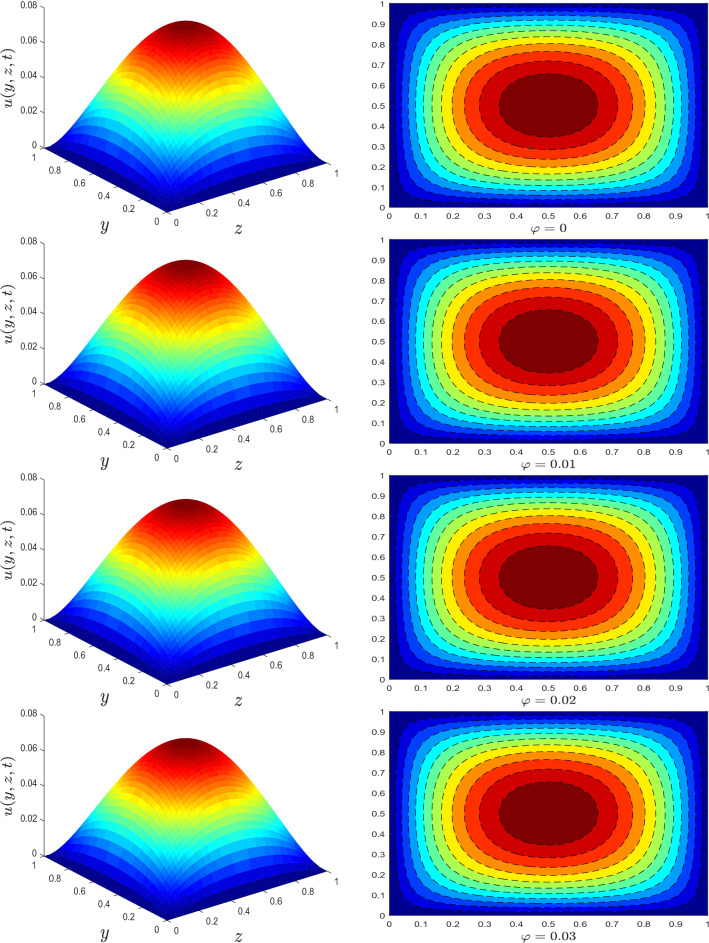
Velocity profile for different values of nanotube volume fraction $\varphi$.

**Figure 4:**
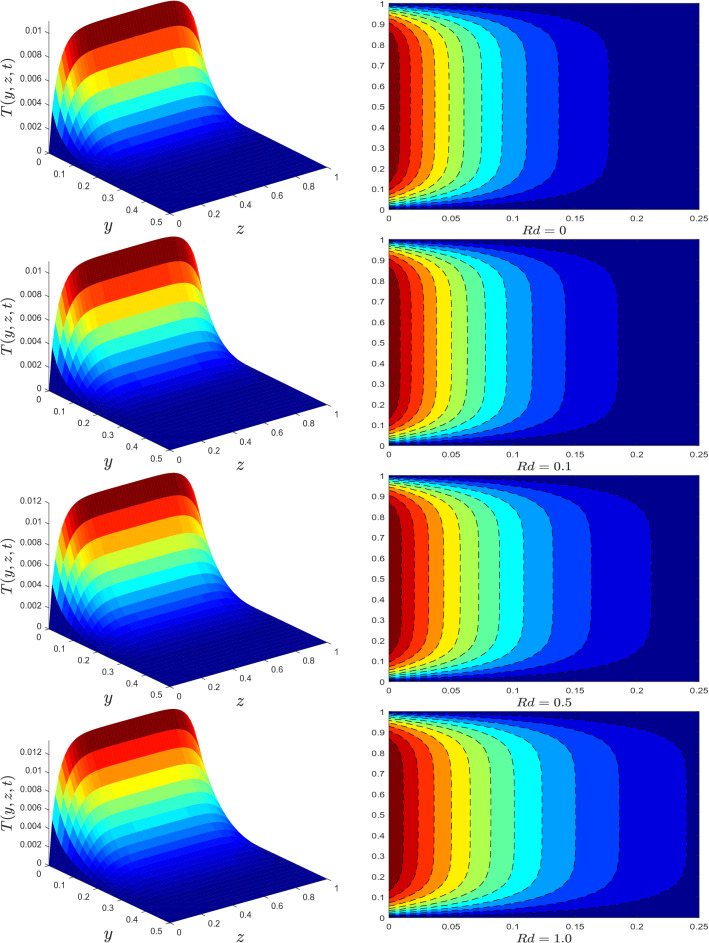
Temperature profile for different values of thermal radiation parameter *Rd*.

**Figure 5:**
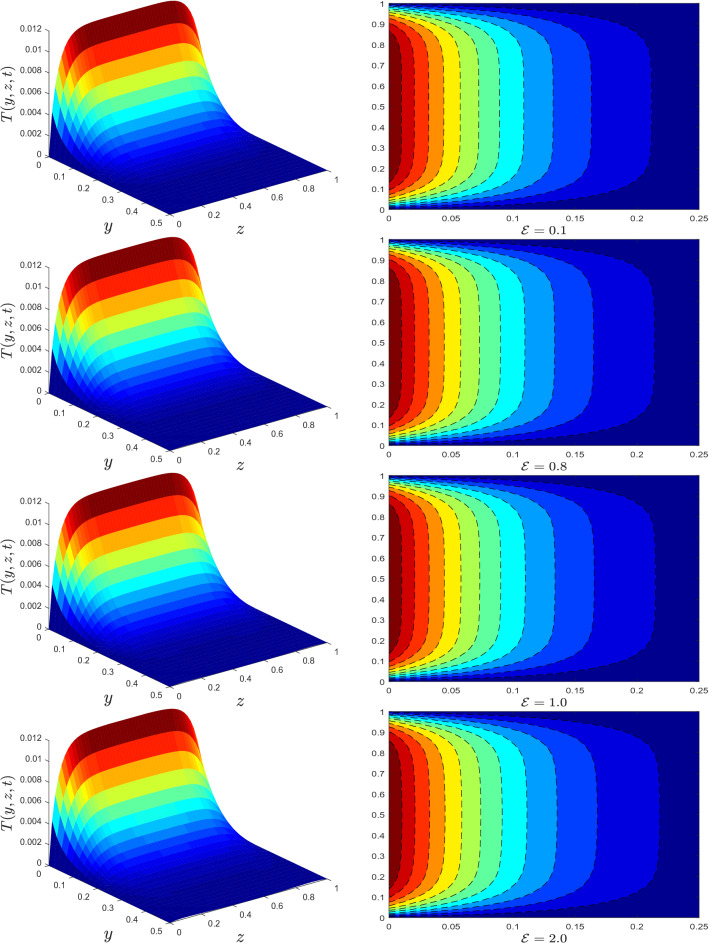
Temperature profile for different values of dissipation parameter ${\mathscr {E}}$.

**Figure 6:**
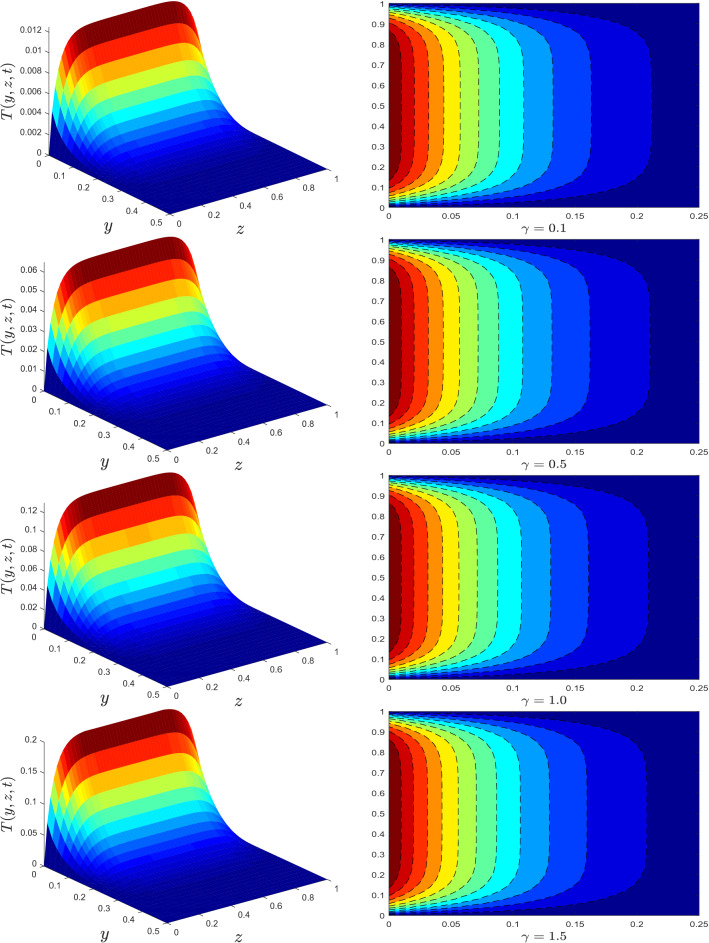
Temperature profile for different values of conjugate heat transfer parameter $\gamma$.

## Results and discussion

Theoretical aspects of MWCNTs on the flow and heat transfer of mineral oil with conjugate boundary conditions will be discussed in this section. The numerical solutions for velocity and temperature are derived using a newly devised Crank–Nicolson finite difference approach in conjunction with L$_1$ algorithm. On flow and heat transfer, the impacts of MWCNTs volume fraction $\varphi$, fractional derivative parameter $\alpha$, relaxation time parameter $\lambda$, radiation parameter *Rd*, viscous dissipation parameter ${\mathscr {E}}$, and conjugate heat parameter $\gamma$ are investigated. The following numerical values for the parameters are assumed to be fixed: $\varphi =0.01, \alpha =0.5, \lambda =0.1, Rd=0.5, {\mathscr {E}}=0.1,$ and $\gamma =0.1$, unless otherwise stated. The parameter ranges shown in the diagrams are as follows: $0\le \varphi \le 0.04, 0.1<\alpha <0.9, 0\le \lambda\le 0.7 , 0\le Rd\le 1.3, 0\le {\mathscr {E}}\le 2, 0.1\le \gamma \le 2$. The thermal physical properties of mineral oil and MWCNTs are provided in Table 1.

The trend of velocity for different parameters is depicted in Figs. 1, 2 and 3. The axial velocity pattern for various fractional derivative parameter $\alpha$ is shown in Fig. 1. It is worth noting that when $\alpha$ gets higher, the amplitude of the velocity gets smaller. Moreover, the obtained surface plot resembles a Gaussian distribution or a traditional heat kernel graph with increasing standard deviation when the fractional derivative power has increased. For larger estimates of relaxation time parameter $\lambda$, an increase in fluid velocity is visualized, see Fig. 2. The influence of nanotube volume fraction $\varphi$ is ascertained in Fig. 3. As illustrated in this graph, the velocity of fluid decreases as the value of $\varphi$ increases. Generally, raising the volume concentration of nanomaterials inside a fluid increases its viscosity. As a result, the velocity of the fluid substantially reduces.

**Figure 7:**
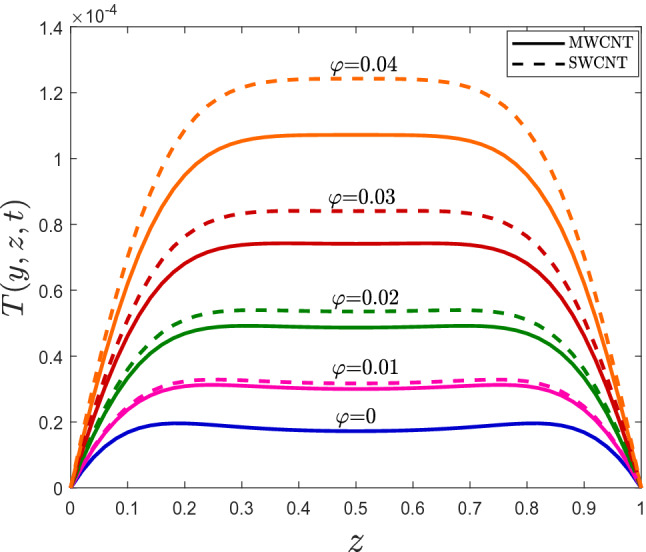
One-dimensional temperature profile for different values of $\varphi$.

**Figure 8:**
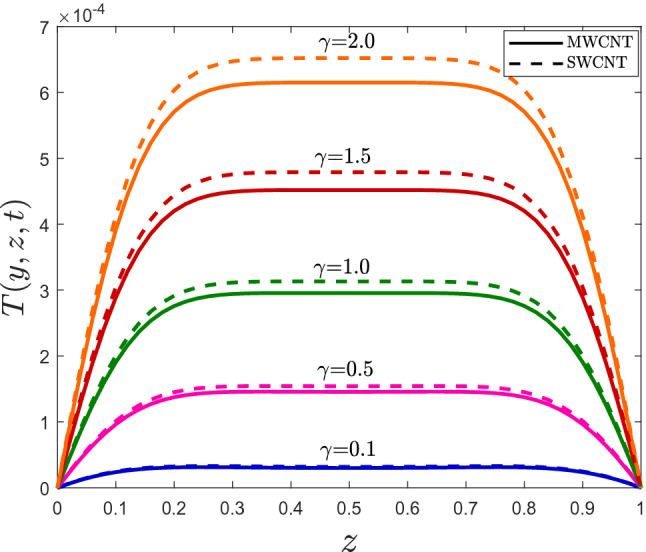
One-dimensional temperature profile for different values of $\gamma$.

**Figure 9:**
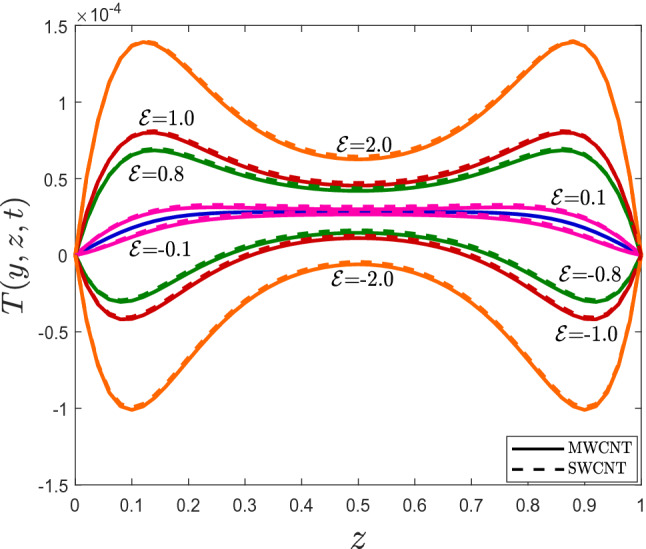
One-dimensional temperature profile for different values of ${\mathscr {E}}$.

The trend of temperature profile for several parameters are illustrated in Figs. 4, 5, 6, 7, 8 and 9. The effects of the thermal radiation parameter *Rd* on the temperature distribution are outlined in Fig. 4. It is observed that, with higher *Rd* values, more heat is generated. As a result, a rise in the fluid’s temperature is perceived. The influence of dissipation parameter ${\mathscr {E}}$ on the temperature distribution is portrayed in Fig. 5. The parameter  ${\mathscr {E}}$ is used to determine the effect of self-heating due to the dissipation properties. At high flow rates, the thermal field in a fluidic framework is swamped by the temperature gradient present in the framework and the effects of dissipation due to internal friction. As seen in Fig. 5, the temperature field is more significant for higher values of ${\mathscr {E}}$. The temperature profile improves because more heat energy is stored in the fluid due to friction forces as ${\mathscr {E}}$ increases. Figure 6 is provided to visualize the effect of conjugate heat parameter $\gamma$ on temperature distribution of Maxwell nanofluid. The temperature is increased when $\gamma$ is increased. Physically, it was expected because more heat is transferred from the heated surface to the cold fluid. As a result, the temperature of the fluid rises.

By fixing the *y*-coordinate in Figs. 7, 8 and 9, a one-dimensional temperature profile is drawn for MWCNTS/mineral oil and SWCNTs/mineral oil. The same conclusions as the surface plots can be drawn; however, the temperature of fluid with SWCNTs is more significant than MWCNTs.

## Conclusions

The mineral oil base nanofluid flow with MWCNTs accompanied by the radiative heat, viscous dissipation and Newtonian heating is deliberated numerically. A finite difference method is used to solve the formulated mathematical problem, and the graphical results are generated in MATLAB software. The following are the most important findings of this research:

- Lower velocity is associated with nanomaterial volume concentration.
- The relaxation time parameter corresponds to higher velocity flow.
- Friction forces generated by the viscous dissipation factor increase the temperature.
- The temperature of Maxwell nanofluid significantly raises against the conjugate heat transfer parameter.

## Greek symbols

$\alpha$: Fractional-order derivative
$\gamma$: Conjugate heat parameter
$$\lambda$$: Relaxation time
$${\mathfrak {h}}$$: Time step
$$\mu$$: Dynamic viscosity
$$\nu$$: Kinematic viscosity
$$\rho$$: Density
$\sigma _{b}$: Stefan Blotzman coefficient
$\varphi$: Nanotube volume fraction
$$\varpi$$: Sphericity

## Roman letters

$${\mathbb {P}}$$: Pressure gradient
${\mathbb {S}}$: Extra stress tensot
$${\mathscr {E}}$$: Viscou dissipation
$$k$$: Thermal conductivity
$$k_{b}$$: Absorption coefficient
$$Rd$$: Thermal radiation
$$T$$: Temperature
$$V$$: Velocity
$${\mathbb {H}}$$: Heaviside function
$${\mathbb {I}}$$: Identitiy matrix
${\mathfrak {p}}$: Mesh size in *y* direction
${\mathfrak {q}}$: Mesh size in *z* direction
$C_{p}$: Specific heat capacity
$$Pr$$: Prandtl number
$q_{r}$: Radiative heat flux
$$t$$: Time

## Subscripts/superscripts

$$f$$: Base fluid
$i$: Regularly spaced grid size in *y* direction
$j$: Regularly spaced grid size in *y* direction
$$k$$: Time level
$$nf$$: Nanofluid
$$s$$: Nanoparticles
